# Pixantrone beyond monotherapy: a review

**DOI:** 10.1007/s00277-019-03749-0

**Published:** 2019-07-17

**Authors:** Cristina Barrenetxea Lekue, Silvina Grasso Cicala, Sirpa Leppä, Thomas Stauffer Larsen, Susana Herráez Rodríguez, Clara Alonso Caballero, Judit M. Jørgensen, Helle Toldbod, Irene Leal Martínez, Francesco D’Amore

**Affiliations:** 1 Hospital Universitario Basurto, Avenida de Montevideo, 18, 48013 Bilbao, Vizcaya Spain; 2 Medical Affairs Department Servier, Madrid, Spain; 3 Department of Oncology, Helsinki University Hospital Comprehensive Cancer Centre and University of Helsinki, Helsinki, Finland; 4 Department of Hematology, Odense University Hospital, Odense, Denmark; 5 Department of Hematology, Aarhus University Hospital, Aarhus, Denmark

**Keywords:** Pixantrone, Non-Hodgkin’s lymphoma, Antineoplastic efficacy, Cardiotoxicity

## Abstract

Outcomes for patients with non-Hodgkin’s lymphoma (NHL) that proves refractory to treatment remain poor. Treatment of such patients is individualized and can include enrolment in a clinical trial of novel agents or use of one of a wide array of drug regimens. Initial treatment with anthracyclines such as doxorubicin limits options at later stages of treatment because of anthracycline-related cumulative cardiotoxicity. The aza-anthracenedione pixantrone was developed to reduce the likelihood of cardiotoxicity without compromising efficacy and is currently conditionally approved for use as monotherapy in patients with multiply-relapsed or refractory aggressive B cell NHL. The use of pixantrone in combination therapy, often to replace doxorubicin or mitoxantrone, has or is currently being investigated in numerous studies in patients with aggressive or indolent NHL and is the focus of this review. These include the R-CPOP regimen (rituximab, cyclophosphamide, pixantrone, vincristine, prednisone) for aggressive NHL in the first-line setting, including a study in elderly patients with limited cardiac function, and for patients with relapsed NHL with prior anthracycline exposure; the PSHAP regimen (pixantrone, cytarabine, prednisone, cisplatin), also in the latter setting; the PREBen/PEBen regimen (pixantrone, bendamustine and etoposide with or without rituximab) as salvage therapy; and pixantrone in combination with fludarabine, dexamethasone, and rituximab (FPD-R) for relapsed indolent NHL.

## Introduction

Pixantrone is a novel aza-anthracenedione, developed to reduce cardiotoxicity typically associated with anthracyclines but without compromising antineoplastic efficacy [1]. Anthracenediones contain only three planar rings versus four of anthracyclines and also lack the daunosamine amino-sugar that anthracyclines possess [2]. Pixantrone has critical differences in its molecular structure compared with the prototypic anthracenedione mitoxantrone, differences that reduce pixantrone’s cardiotoxic potential compared with mitoxantrone [2]. In pixantrone, a hydroquinone was removed and a nitrogen heteroatom inserted in the same ring, and (ethylamino)diethylamine side chains were substituted for (hydroxyethylamino)ethylamino side chains (as reviewed by Menna and colleagues [2]).

Its mechanism of action includes anthracycline-like DNA alkylation and formation of permanent double-strand breaks and resultant apoptosis, but recent lines of evidence suggest a more prominent role for sequential rounds of aberrant mitosis leading to cell death [2]. Unlike anthracyclines and anthracenediones, it is only a weak inhibitor of topoisomerase II [3]. Pixantrone’s novel molecular structure aimed to eliminate interactions with iron, thus reducing its potential for cardiotoxicity typically associated with anthracyclines [2]. Pre-clinical study results of pixantrone monotherapy suggested less cardiotoxicity compared with doxorubicin and mitoxantrone [4–6].

The European Medicines Agency issued a conditional marketing authorization for pixantrone in May 2012 based on available clinical evidence [7]. The phase III trial of pixantrone monotherapy (“PIX301”) in 140 adult patients with relapsed/refractory aggressive non-Hodgkin’s Lymphoma (NHL) confirmed its antineoplastic efficacy and clinically acceptable toxicity profile [8, 9]. Complete response (CR)/unconfirmed CR (uCR) [primary endpoint] and overall response rates (ORRs) were significantly higher with pixantrone monotherapy than physician’s choice comparator chemotherapy regimens: 20% vs 5.7% (*p* = 0.021) and 37.1% vs 14.3% (*p* = 0.003), respectively [8]. Progression-free survival (PFS) was significantly longer with pixantrone (5.3 vs 2.6 months; *p* = 0.005) [8]. Post hoc analyses of the subpopulation of patients with relapsed/refractory aggressive B cell NHL (*n* = 97) confirmed the advantage of pixantrone over comparator regimens, and this advantage was maintained in patients receiving pixantrone as third- or fourth-line therapy irrespective of previous rituximab treatment (with rituximab: pixantrone vs comparator ORR of 45% vs 11.1% *p* = 0.033 and PFS 5.4 vs 2.8 months, hazard ratio [HR] 0.52, 95% confidence interval [CI] 0.26–1.04; without rituximab: ORR of 42.1% vs 14.3%, *p* = 0.078; PFS 6.1 vs 3.5 months, HR 0.36, 95%CI 0.18–0.73) [9]. Pixantrone tolerability in the B cell subpopulation post hoc analysis was concordant with results from the overall patient population, and the frequency of cardiac adverse events (AEs) did not increase with increasing pixantrone exposure [9]. Another post hoc analysis of the PIX301 study investigated any possible correlations between patient characteristics and clinical response in the 17 patients (median age 61 years; 58.8% with diffuse large B cell lymphoma [DLBCL]; 70.6% had received two prior lines of therapy) treated who achieved a CR or uCR with pixantrone in the study (24% of patients). While the majority of patients (64.7%) who achieved a CR in this analysis had responded (CR/partial response [PR]) to the previous line of treatment, the achievement of a durable response appeared to be independent of the type of response to prior therapy [10].

Pixantrone is approved for use as monotherapy in adult patients with multiply-relapsed or refractory aggressive B cell NHLs [3]. It is administered intravenously on days 1, 8, and 15 of a 28-day cycle for up to six cycles at a dosage of 50 mg/m^2^ (base form dose) [3]. Intravenous pixantrone offers a predictable pharmacokinetic profile [11]. It displays linear pharmacokinetics over the 3–105 mg/m^2^ dose range [3]. After intravenous administration, distribution is rapid, with a prolonged elimination phase (mean half-life approximately 23 h) [3]. It has a large volume of distribution (25.8 L) and is approximately 50% plasma protein-bound [3]. Drug metabolism is limited; its primary route of excretion is as unchanged drug in the bile [3].

Patients with indolent or aggressive NHL who are refractory to treatment or who experience multiple relapses remain difficult to treat. Novel treatment regimens are required, and on this basis, pixantrone has been investigated as combination therapy in clinical studies in both aggressive [12–17] and indolent [18, 19] forms of NHL. The aim of this narrative review is to describe the current evidence for the use of pixantrone in combination therapy in refractory/relapsed aggressive or indolent NHLs, and to review ongoing studies of pixantrone in this setting. As this is a narrative review, there was no structured search strategy.

## Pixantrone in aggressive lymphomas

### CPOP regimen

R-CHOP (rituximab, cyclophosphamide, doxorubicin, vincristine, prednisone), with or without radiotherapy, is a standard regimen for the first-line treatment of DLBCL, although the specific type of R-CHOP regimen may vary according to clinical need [20, 21]. In an effort to minimize anthracycline-related cardiotoxicity, pixantrone (150 mg/m^2^ intravenously) was used in place of doxorubicin (R-CPOP, 21-day cycle) in a comparative, phase II, open-label study in 124 untreated adult patients with CD20^+^ DLBCL [14]. Efficacy results showed that the R-CPOP regimen (*n* = 61) is an active regimen and safety results showed substantially lower cardiotoxicity than with R-CHOP (*n* = 63) [14]. The CR/uCR response rate was 75% with R-CPOP versus 84% with R-CHOP. While the study was intended to be a non-inferiority study, enrolment was halted early due to regulatory constraints and thus the study was underpowered to confirm the non-inferiority of R-CPOP versus R-CHOP. Other efficacy endpoints were determined as supportive evidence and were similar between groups: for example, median PFS was not reached in the R-CPOP treatment arm probably due to premature study termination and was 40 months in the R-CHOP arm (HR 1.02; 95%CI 0.60–1.76; *p* = 0.934) [14]. The most common AEs (≥ 10%) in both treatment arms were general and hematologic disorders and the most common grade 3/4 drug-related AEs in both treatment groups were neutropenia, leukopenia, lymphopenia, febrile neutropenia, and anemia. The proportions of patients with congestive heart failure (CHF) [0% vs 6%; *p* = not significant], a decline from baseline in ejection fraction (EF) of ≥ 20% (2% vs 17%; *p* = 0.004), and elevations in troponin-T levels (7% vs 33%; *p* = 0.003) were lower in the R-CPOP than R-CHOP group [14]. While non-inferiority was not demonstrated, the cardiotoxicity results seem to support the pre-clinical characteristics of pixantrone as compared with doxorubicin. Study authors concluded that their results support further investigation of pixantrone as first-line therapy in high cardiac risk DLBCL patients [14].

Indeed, there is an ongoing open-label phase II study in Germany and Austria of first-line R-CPOP in elderly patients with DLBCL including in those with limited cardiac function [EudraCT number: 2014-005069-60) [22]. Adults with DLBCL or grade IIIB follicular lymphoma (FL) will be enrolled, in two pre-specified subpopulations: (1) elderly patients (≥ 75 years) not eligible for standard R-CHOP21 and (2) patients with impaired cardiac function (EF ≥ 40% and ≤ 50%). Preliminary results from one of the centers participating in the study (the University Hospital Freiburg, Germany [*n* = 10]) suggests this regimen is feasible and well tolerated with expected hematological toxicities and no neutropenia-related deaths [17]. Eight patients had DLBCL, one had high-grade B cell lymphoma, and one had Richter transformation; their median age was 72.4 years (range 61–84) and all patients had clinical CHF at baseline. Eight patients completed four to six cycles of R-CPOP, of whom none experienced higher-grade acute cardiac toxicity while on treatment [17]. Early efficacy results showed confirmed complete remission in five patients and current median overall survival (OS) of 10 months (range 2–31 months) [17].

The use of an anthracycline-based regimen such as R-CHOP in second-line treatment is limited by cumulative anthracycline-related cardiotoxicity [12]. Pixantrone is suited for use therefore in patients with relapsed NHL with prior anthracycline exposure. Borchmann and colleagues assessed the tolerability and potential efficacy of CPOP in a phase I/II study in adult patients (*n* = 35/30) with relapsed aggressive NHL who had previously received CHOP (with or without rituximab) and who were ineligible for stem cell transplantation (SCT) [12]. The majority of patients had DLBCL or grade III FL. Pixantrone was given at the dose determined by the phase I part of the study (150 mg/m^2^) in combination with fixed standard doses of cyclophosphamide (750 mg/m^2^ IV) and vincristine (1.4 mg/m^2^ IV, not exceeding 2.0 mg), all of which were administered on day 1. Prednisone or prednisolone (100 mg orally) was administered on days 1 through 5 in each of and up to six 21-day cycles. This regimen gave a CR/uCR of 47% and a median CR duration of 10.5 months [12]. The ORR was 73%, and median OS was 17.9 months [12]. In the phase II part, serious AEs of grade 3 or 4 severity were hematologic AEs (70% of patients) and infections (23%), and febrile neutropenia occurred in 20% of patients [12]. Small study size precluded authors from making definitive conclusions about the cardiac safety of this regimen, and pre-existing conditions confounded determination of causality in four patients who developed symptomatic heart failure [12]. Study authors noted that their patients already had a mean prior doxorubicin-equivalent drug exposure of about 300 mg/m^2^ at baseline and most then went on to receive six cycles of CPOP. They suggested that the rate of clinically significant cardiac AEs in their study could be considered lower than expected than if their patients had instead received six cycles of CHOP [12], based on analyses of the relationship between doxorubicin cumulative dose and doxorubicin-related CHF [23].

Recent Spanish (“GELTAMO”) guidelines for the treatment of patients with DLBCL mention that the results of this CPOP trial are noteworthy when considering options for second-line therapy in patients with relapsed DLBCL [24].

### PREBEN/PEBEN regimen

Bendamustine and etoposide may be ideal candidate drugs for use in combination with pixantrone (PEBEN) as they may act synergistically with pixantrone [2]. This combination (with rituximab in CD20^+^ tumors [PREBEN]) originally developed by the Nordic Lymphoma Group (NLG) was first reported in 2014 [25] and consisted of pixantrone 50 mg/m^2^ (base form dosage) on day 1 and day 8 plus etoposide 100 mg/m^2^ on day 1 plus bendamustine 90 mg/m^2^ on day 1, with or without rituximab 375 mg/m^2^ on day 1 of a 21-day cycle (maximum 6 cycles) [13, 26].

The NLG has also used this combination (with rituximab in CD20^+^ tumors [PREBEN]) in 30 heavily pretreated patients with aggressive NHL (Table 1; data available as a poster) [13], with their findings informing subsequent clinical trial design, as well as by a Spanish group in five patients with refractory or relapsed DLBCL [26]. These early results suggest that PREBEN/PEBEN is a feasible salvage regimen, with durable and substantial responses to treatment in individual patients (Table 2) [13]. Additionally, there were differences in response in the DLBCL subgroup between multiple-relapse patients and patients with refractory disease: seven of nine “frail” patients with relapsed disease not eligible for transplant and one of two patients with relapses post-transplant responded to treatment whereas only one of six primary refractory patients responded. In addition, PREBEN/PEBEN acted as bridging therapy in patients with peripheral T cell lymphoma [PTCL] (4 of 7 proceeded to non-myeloablative allogeneic transplant) [13]. Similarly, positive success using PREBen/PEBen as a bridging therapy was obtained in three of five (60%) patients at the Spanish center who received previous chemotherapy with multiple drugs (mean lines of treatment = 3), who achieved objective responses after two treatment cycles, and two of these patients experienced a CR making them eligible for an allogeneic transplant after cycle 6 [26].

**Table 1:** Summary of baseline patient characteristics in preliminary clinical experiences in Europe of the PREBen salvage regimen in patients with relapsed aggressive B- and T cell lymphomas

	Observational study [13]	Ongoing phase I/II trial [27]
No. of pts	30	12
Male/female	19/11	8/4
Age range, year	49–81	39–80
No. of previous chemotherapy regimens, mean (range)	3 (1–7)	3 (1–5)
Prior ASCT, *n*	NR	3
Histopathology, *n*		
DLBCL	17	8
tIND	6	–
PTCL	7	4
IPI score > 2, *n*	30	12

*ASCT* autologous stem cell transplant, *DLBCL* diffuse large B-cell lymphoma, *IPI* International Prognostic Index, *NR* not reported, *PREBen* pixantrone, bendamustine, etoposide, rituximab, *PTCL* peripheral T cell lymphoma, *tIND* transformed indolent lymphoma

**Table 2:** Summary of preliminary efficacy results of the PREBen salvage regimen in patients with relapsed aggressive B- and T cell lymphomas

	Observational study ([13]) *N* = 30	Ongoing phase I/II trial ([27]) *N* = 10^a^
CMR, *n* (%)	8 (27)	4 (40)
PMR, *n* (%)	7 (23)	6 (60)
ORR, *n* (%)	15 (50)	10 (100)
^b^Response duration, month	2–23+	4–7+

*CMR* complete metabolic response, *ORR* overall response rate, *PMR* partial metabolic response, *PREBen* pixantrone, bendamustine, etoposide, rituximab

^a^After 2 cycles of treatment

^b^Response duration data are indicative only based on time point at which data were analyzed, rather than formal study end. + sign indicates response was ongoing at the data cut-off point

Given these encouraging results, this combination is now being investigated in a NLG-coordinated intergroup trial in collaboration with the HOVON group. This is an open-label phase I/II trial investigating PREBEN/PEBEN as a salvage regimen for heavily pretreated patients with aggressive B or T cell lymphomas (NCT02678299, EudraCT no. 2015-000758-39) [16, 27]. The phase I part of the study has now been completed [16]. Patient characteristics prior to salvage PREBEN/PEBEN are summarized in Table 1. The majority had DLBCL and all had intermediate or high IPI risk scores. According to pre-defined criteria, patients were subdivided into “fit” and “frail” groups: the former entered the dose-finding phase I part at baseline dose level and frail patients enter the phase II part directly, also at the same baseline dose level [16]. The baseline treatment regimen was the same as the regimen in the compassionate use study but with a maximum of 4 to 6 cycles [16].

Interim efficacy results have been reported for the first 10 patients (in phase I or II) and are summarized in Table 2, indicating that PREBEN/PEBEN is a feasible salvage regimen, even after only a few treatment cycles [27]. All 10 patients experienced a response to treatment and 40% had a complete metabolic response (CMR) [27].

Five patients completed phase I, and according to the primary endpoint, the maximum tolerated dose of PREBEN is pixantrone 50 mg/m^2^ day 1 and 8, etoposide 100 mg/m^2^ on day 1, bendamustine 90 mg/m^2^ on day 1, and rituximab 375 mg/m^2^ on day 1 [16]. The two dose-limiting toxicities were neutropenic infection and post-therapeutic neutropenia (< 0.5 × 10^9^/L) for more than 5 days in a patient without marrow involvement [16].

Considering the available evidence, the PREBEN/PEBEN salvage regimen seems to be generally well tolerated. In the observational study [13], 52% of patients experienced grade 3–4 hematologic toxicities (mainly neutropenia and thrombocytopenia) and 21% grade 3–4 infections. One patient with PTCL developed CHF but had prior doxorubicin exposure. One patient with transformed indolent lymphoma (tIND) developed acute myeloid leukemia with therapy-related cytogenetic features but had previously received ibritumomab tiuxetan. In the phase I/II study, preliminary safety data for all 10 patients at the time of the analysis confirmed the most common grade 3–4 toxicity to be hematologic, grade 3–4 infections were reported in two patients [27]. The Spanish-center experience confirmed no new unexpected AEs with PREBEN [26].

### PSHAP regimen

The ESHAP (methylprednisone, etoposide, cytarabine, cisplatin) regimen has been commonly used in the setting of relapsed/refractory aggressive NHL [15]. Current National Comprehensive Cancer Network (NCCN) guidelines include this regimen among others (with or without rituximab) for patients with relapsed or refractory DLBCL that is chemosensitive at relapse and who are candidates for high-dose therapy/autologous stem cell rescue (HDT/ASCR) [21].

**Fig. 1:**
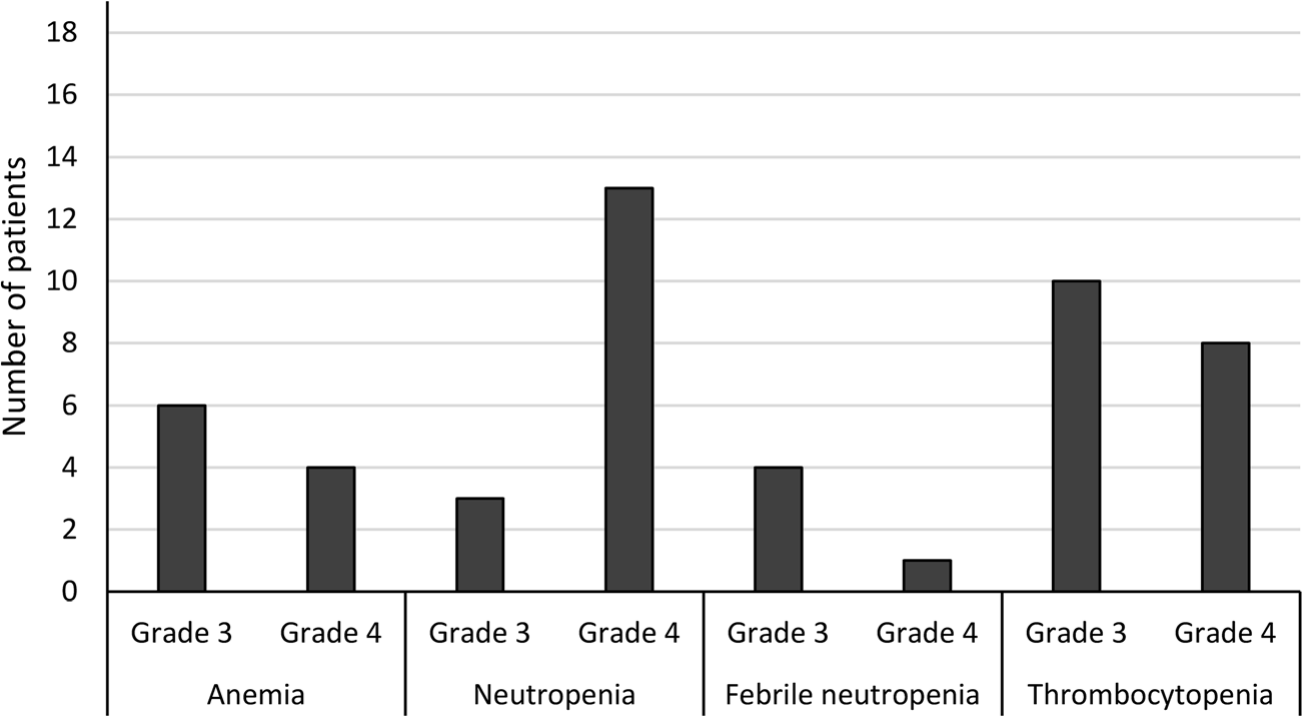
Tolerability of PSHAP (pixantrone, cytarabine, cisplatin, prednisone) regimen in 19 patients with relapsed/refractory aggressive non-Hodgkin’s lymphoma in a phase I/II dose-escalation study [15]

In a phase I/II study in 19 adult patients with relapsed/refractory aggressive NHL (mainly DLBCL) all of whom had received prior doxorubicin, pixantrone replaced etoposide in the ESHAP regimen (PSHAP) [15]. The PSHAP regimen was pixantrone 80 mg/m^2^ (base form dose) on day 1 plus, at fixed doses, cytarabine 2000 mg/m^2^ on day 5, prednisone 500 mg/m^2^ days 1–5, and cisplatin 25 mg/m^2^ on days 1–4 of a 21 day cycle [15]. All drugs were administered intravenously. Efficacy results were particularly promising after a median of four treatment cycles (CR 37%, PR 21%, ORR 58%) [15]. Six of 11 patients responding to PSHAP underwent SCT, and thus, this regimen should be evaluated further for reducing pre-transplant tumor burden [15]. Hematologic toxicities were considered to be clinically acceptable (Fig. 1), and only one patient developed febrile neutropenia, and none died from infection or hemorrhage [15]. No clinically significant cardiac toxicity occurred. Seven patients had left ventricular ejection fraction (LVEF) decreases from baseline (although none ≥ 19% vs baseline), which were transient in three patients [15].

### Pixantrone plus rituximab

The efficacy and safety of pixantrone plus rituximab (PIX + R) was compared with gemcitabine plus rituximab (GEM + R) in patients with aggressive relapsed or refractory B cell NHL in the randomized, multicenter, phase III PIX 306 trial (NCT01321541) [28, 29]. This trial was initiated to meet post-authorization requirements because the pivotal registrational trial of pixantrone monotherapy [8] had been designed before rituximab became the standard of care [7, 28]. The trial enrolled 312 patients (median age 73 years; range 26–91) in whom the primary efficacy endpoint of PFS was not reached. The median PFS of 7.3 months in the PIX + R group versus 6.3 months in the GEM + R group. The median OS (13.3 vs 19.6 months) and the ORR (61.9% vs 43.9%) was also higher in the PIX + R group compared with GEM + R group, which also had a higher proportion of patients achieving a complete response (35.5% vs 21.7%) [29].

## Pixantrone in relapsed or refractory indolent lymphomas

There is large diversity in the spectrum of NHL subtypes [30]. While an indolent course is described for a number of different subtypes, the most common subtype of indolent NHL is FL, which accounts for 22% of all newly diagnosed cases of NHL [31]. Most patients with indolent lymphomas eventually die after a chronically relapsing and remitting disease course [32]. There are many therapeutic options for patients with relapsed FL: rituximab monotherapy, combination chemotherapy plus rituximab, SCT (where appropriate), and radioimmunotherapy [33]. Chemotherapy options include multi-agent regimens that are platinum salts-, alkylating agents-, or fludarabine-based [34].

Although not approved for use in patients with relapsed/refractory indolent NHL, pixantrone has been studied in this setting in combination with rituximab in a phase III trial [18] and in combination with fludarabine, dexamethasone, and rituximab in a phase I study (FPD-R) [19]. The focus of this section is on the latter dose-escalating study with an expansion cohort, which assessed the safety and efficacy of FPD-R [19]. At the time this study was designed, FND (fludarabine, mitoxantrone, and dexamethasone) was a standard regimen for relapsed or refractory indolent NHL [19]. The rationale for replacing mitoxantrone with pixantrone was pixantrone’s more favorable cardiac toxicity profile in pre-clinical models [35], and its efficacy in relapsed or refractory aggressive NHL [8].

The FPD-R regimen was shown to be highly active and well tolerated in relapsed indolent NHL in a phase I dose-escalation study [19]. Most patients enrolled in this trial had follicular center cell lymphoma or small lymphocytic lymphoma. Patients received a median of 5 28-day cycles [19]. Of 27 evaluable patients, the ORR was 89%, the CR 63%, uCR 7%, and PR 19%. Treatment response was durable, with a median response of 23 months. The most common toxicities were hematologic, but there was a low incidence of febrile neutropenia (4%). Treatment-related serious AEs were reported in 14 patients and 9 patients discontinued study treatment because of AEs (hematologic AEs [*n* = 4], grade1–2 decrease in LVEF [n = 4], rash [*n* = 1; related to rituximab]) [19]. However, there were no grade 3–4 cardiovascular AEs or episodes of CHF. Seven of eight patients with grade1–2 decreases in LVEF had previously received anthracyclines [19].

These promising safety and efficacy results of pixantrone combination therapy for relapsed indolent NHL are supported by results from the randomized, open-label phase III trial (*n* = 38) [data available as an abstract] [18]. Although the study was discontinued earlier than expected due to low patient enrollment, the results suggested that pixantrone plus rituximab may be more effective than rituximab monotherapy in this patient population (ORR 75% vs 33%, CR 35% vs 11%, PR 40% vs 22% and time to progression: 13.2 vs 8.1 months) [18]. The combination was generally well tolerated, with only two drug-related serious AEs (febrile neutropenia [*n* = 2]) although six patients in the combination group withdrew because of AEs (none did so in the rituximab arm) [18].

## Safety and tolerability of pixantrone

Pixantrone is reported to have significant antineoplastic efficacy with lower cardiotoxicity compared with other anthracyclines [1]. Several mechanisms have been proposed for its limited potential to produce cardiomyocyte damage, including its inability to bind iron [36], and its greater selectivity for topoisomerase IIα than IIβ [37]. Evidence to date from pixantrone monotherapy trials (as reviewed previously [35]) and the combination therapy trials reviewed here tend to confirm an acceptable safety profile in terms of cardiotoxicity. However, it remains important to continue to assess cardiotoxicity in all future trials and particularly with post marketing surveillance, which may detect “late” cardiotoxicity [38]. Results from new studies specifically including patients with pre-existing cardiac dysfunction (e.g., the first-line R-CPOP study [22]) are awaited with interest in this regard.

Like other cytotoxic chemotherapies, myelosuppression is very common with pixantrone [8, 38], and this remains the case when given in combination therapy. Overall, there appear to be no new or unexpected AEs when pixantrone is given in combination therapy.

## Other ongoing pixantrone combination therapy trials

There are three ongoing studies of interest: a phase I/II trial of pixantrone in combination with bendamustine and rituximab in patients with relapsed/refractory aggressive B cell NHL (NCT01491841) [39]; two non-comparative, open-label, multicenter phase II trials, the first in patients with relapsed aggressive B cell lymphoma are being treated with pixantrone plus the novel type II anti-CD20 antibody obinutuzumab (GA101) [“GOAL”; NCT02499003] [40, 41] and the second in CD20^+^ patients with relapsed or refractory aggressive NHL who will receive pixantrone in combination with ifosfamide, etoposide, and rituximab (NCT03458260) [42]. Preliminary results are available from the multicenter, non-randomized, investigator-initiated GOAL trial that enrolled 67 patients (median age 75 years, 55.5% female) who were treated with up to 6 cycles of pixantrone 50 mg/m^2^ on days 1, 8, and 15 of each cycle and obinutuzumab 1000 mg flat dose on day 1, 8, and 15 of cycle 1 and day 1 of each subsequent cycle. This study reported a median PFS of 82 days after a median follow-up of 8.2 months; the median OS was not reached (both secondary endpoints) [43].

## Discussion and conclusions

Treatment challenges remain in the management of relapsed aggressive NHL as a there is a lack of regimens as effective as first-line therapy [11]. Results from the SCHOLAR-1 retrospective study (*n* = 636), representing the largest number of patients with refractory DLBCL included in an analysis of response and survival rates in the rituximab-era confirms the poor long-term outcomes of these patients (ORR 26% and median OS 6.3 months) [44].

Pixantrone was effective as monotherapy in multiply-relapsed/refractory aggressive NHL, according to results from the PIX301 trial [8], and may be associated with durable response and long-term remission in some patients, according to a recent post hoc analysis of data from this trial (*n* = 17) [10]. On the basis of the results of the PIX306 trial [29], the European Medicines Agency (EMA) has recently issued a positive opinion for pixantrone and converted its conditional approval into a standard marketing authorization as a monotherapy for the treatment of adult patients with multiply-relapsed or refractory aggressive non-Hodgkin B cell lymphoma [45]. While patients may remain sensitive to anthracyclines, which are often used in first-line therapy, cumulative dose toxicity limits their use in subsequent lines of therapy [11], and this is where pixantrone has the potential to transform the management of patients with relapsed aggressive disease. Multi-agent pixantrone-containing regimens such as CPOP, PREBen/PEBEN, and PSHAP, as reviewed here, seem to offer feasible alternatives to commonly used regimens in the multiply-relapsed setting with no new or unexpected AEs, although data for PREBen/PEBen are very preliminary and further comparative prospective phase II/III trials with these regimens would be informative.

Focus has shifted to whether pixantrone in R-CPOP may be a novel option in the first-line setting for aggressive NHL, particularly in patients with cardiac impairment or in those not eligible for standard first-line R-CHOP21, and a phase II trial has been initiated [22]. Data from a previous study suggests R-CPOP is better tolerated than R-CHOP, particularly in terms of cardiotoxicity, and this study had included patients with a history of cardiac events [14]. Thus, pixantrone may be particularly useful for patients who have pre-existing comorbidities which would preclude use of anthracyclines [11].

Pixantrone has also been used in relapsed/refractory indolent lymphoma as part of the FPD-R regimen [19] and in combination with rituximab [18] and was shown to be highly active and well tolerated. Given that fludarabine-based regimens are included among the dual/multi-agent regimens recommended as salvage treatment for patients with relapsed FL, the combination of pixantrone with rituximab and fludarabine seems worthy of investigation [34]. Likewise, pixantrone could replace mitoxantrone in FCM-R (fludarabine, cyclophosphamide, mitoxantrone-rituximab) in this setting.

In conclusion, there is accumulating evidence for the potential efficacy and safety of pixantrone for use in combination regimens for the management of relapsed/refractory aggressive or indolent NHLs. Several clinical trials are ongoing, one of which will also investigate pixantrone as first-line therapy in the R-CPOP regimen, including in a subgroup of patients with impaired cardiac function. As results of these trials become available, the place of pixantrone as a valuable component of combination therapy for NHL treatment may become more firmly established.
